# Creativity in hand hygiene program

**DOI:** 10.1186/1753-6561-5-S6-P125

**Published:** 2011-06-29

**Authors:** ML Ling, KB How, KY Tan, LC Lee

**Affiliations:** 1 Infection Control, Singapore General Hospital, Singapore, Singapore

## Introduction / objectives

During SARS, high compliance in healthcare workers to hand hygiene was primarily driven by fear.However, the post-SARS period confirmed that this practice was not sustainable.At the Singapore General Hospital, a 1600-bedded acute tertiary care hospital, the hand hygiene program was revised in late 2006 following Singapore's signing of the pledge to the WHO "Clean Care is Safer Care" program.

## Methods

The WHO audit tool was used in the measurement of hand hygiene compliance from 2007. Innovative education and prominent cues to action were initial building blocks used in transforming behaviour. Creative use of posters, stickers, advertisements on shuttle buses help to create awareness in staffs, patients and the public. A giant poster placed on one of the hospital walls is a strong public statement expressing the hospital's commitment to patient safety through good hand hygiene practices. Incentives in the form of handphone straps, magnets, bookmarks and vouchers given to staffs are other innovative means of staff engagement. The program was enhanced further with system changes, evaluation and feedback; and the fostering of institutional safety climate. Annually, the WHO Hand Hygiene day on 5 May was observed with creative fun activities involving both staffs and public.

## Results

Hand hygiene compliance rate improved from 20% (2007) to 61% (2010). Improvement was also seen annually in the compliance to each of the 5 moments as well as in all staff categories. Healthcare-associated MRSA infections was reduced from 0.6 (2007) to 0.3 (2010) per 1000 patients days.

## Conclusion

Leadership’s support of the program through its visible presence, messaging and release of resources is the key factor in helping to make the program a true success. The hospital was recognised as a Global Hand Hygiene Expert Centre in January 2011.

## Disclosure of interest

None declared.

